# Single nucleotide polymorphisms (SNPs) distinguish Indian-origin and Chinese-origin rhesus macaques (*Macaca mulatta*)

**DOI:** 10.1186/1471-2164-8-43

**Published:** 2007-02-07

**Authors:** Betsy Ferguson, Summer L Street, Hollis Wright, Carlo Pearson, Yibing Jia, Shaun L Thompson, Patrick Allibone, Christopher J Dubay, Eliot Spindel, Robert B Norgren

**Affiliations:** 1 Genetics Research and Informatics Program, Oregon National Primate Research Center, Oregon Health and Sciences University, Beaverton, OR 97006, USA; 2 Washington National Primate Research Center, University of Washington, Seattle, WA 98195, USA; 3 Department of Genetics, Cell Biology and Anatomy, University of Nebraska Medical Center, Omaha, NE 68198, USA

## Abstract

**Background:**

Rhesus macaques serve a critical role in the study of human biomedical research. While both Indian and Chinese rhesus macaques are commonly used, genetic differences between these two subspecies affect aspects of their behavior and physiology, including response to simian immunodeficiency virus (SIV) infection. Single nucleotide polymorphisms (SNPs) can play an important role in both establishing ancestry and in identifying genes involved in complex diseases. We sequenced the 3' end of rhesus macaque genes in an effort to identify gene-based SNPs that could distinguish between Indian and Chinese rhesus macaques and aid in association analysis.

**Results:**

We surveyed the 3' end of 94 genes in 20 rhesus macaque animals. The study included 10 animals each of Indian and Chinese ancestry. We identified a total of 661 SNPs, 457 of which appeared exclusively in one or the other population. Seventy-nine additional animals were genotyped at 44 of the population-exclusive SNPs. Of those, 38 SNPs were confirmed as being population-specific.

**Conclusion:**

This study demonstrates that the 3' end of genes is rich in sequence polymorphisms and is suitable for the efficient discovery of gene-linked SNPs. In addition, the results show that the genomic sequences of Indian and Chinese rhesus macaque are remarkably divergent, and include numerous population-specific SNPs. These ancestral SNPs could be used for the rapid scanning of rhesus macaques, both to establish animal ancestry and to identify gene alleles that may contribute to the phenotypic differences observed in these populations.

## Background

The rhesus macaque (*Macaca mulatta*) has served a critical role in the study of human disease for more than half a century. This macaque remains the animal of choice for much of biomedical research and is the primary model for the study of human immunodeficiency virus (HIV) and acquired immune deficiency syndrome (AIDS) [1]. Though Indian-origin rhesus were originally used in most research protocols, the 1978 ban on the export of primates from India resulted in reduced availability of these animals. Because the growing demand for rhesus macaques has exceeded the domestic supply, the U.S. breeding colonies have imported large numbers of these animals from China.

In recent years, a variety of studies have investigated the relationship between Indian and Chinese rhesus macaques. Comparisons of Indian and Chinese rhesus mitochondrial DNA (mtDNA) sequences, including the *hypervariable sequence I *(*HVS1*), *12S *and *16S rRNA *loci, have shown that as much as 90% of the mtDNA genetic heterogeneity is accounted for by country of origin differences [2-4]. Studies of chromosomal microsatellite loci have also identified marked differences in allele frequencies between Indian and Chinese rhesus macaque populations [5-8]. Similarly, population-specific differences in the allele distributions within both Class I and II major histocompatibility complex (MHC) loci support the contention that the two populations have distinct genetic characteristics [9,10]. All of these studies support the conclusion that since their geographic separation, Indian and Chinese rhesus macaques have diverged to become two separate subtypes.

The genetic divergence of Indian and Chinese rhesus macaques is thought to underlie the observed phenotypic differences between the two subtypes in experimental protocols. These differences are observed at many levels, including morphology, behavior and physiology [11,12]. Significant differences in host response and disease progression have been observed in Indian and Chinese rhesus macaques exposed to the same simian immunodeficiency virus (SIV) challenge [13,14]. Both viremic peaks and set points were, in general, lower in the Chinese rhesus macaques than in Indian animals exposed to SIV/DeltaB670, leading to much longer survival in the Chinese animals [14]. The Chinese rhesus macaques are also more resistant to SIVmac239, maintaining both lower acute and chronic viral loads than Indian rhesus macaques infected with the same viral challenge [13].

Because Indian and Chinese rhesus macaques can respond very differently to the same research protocol, consideration of animal ancestry is warranted in both study design and result interpretation. Most breeding facilities in the United States include both Indian and Chinese-origin rhesus. Although efforts are taken not to interbreed the two populations, lack of information or misinformation regarding animal history can result in unrecognized crossing of the two subtypes. The introduction of misclassified animals may compromise research results. Thus, being able to correctly identify rhesus macaque ancestry, including hybrids, would be very valuable to both researchers and animal breeders.

Current approaches for rhesus macaque ancestry determination include: 1. sequence comparison of mtDNA loci, [2,7]; 2. microsatellite analysis [6,7]; and 3. SNP analysis [15]. Since mtDNA is only inherited through the maternal line, this analysis cannot identify Indian-Chinese hybrid animals. The microsatellite analysis involves three loci in which the allele frequencies differ significantly between Indian and Chinese populations. The current literature only reports five SNPs in 3 genes (*NDN*, *H19 *and *IGF2*) that are unique to either Indian or Chinese rhesus macaques, identified by Fujimoto [15]. Unfortunately, though both the reported microsatellite and SNP markers can work for distinguishing purebred Indian and Chinese rhesus, neither offers a sufficient number of markers to reliably detect hybrid animals.

We have developed algorithms for identifying primers to amplify the 3' end of all rhesus macaque genes [16]. Over 5,000 of the sequences we obtained using these primers were used in the design of the Affymetrix rhesus macaque GeneChip [16,17]. We tested whether these primers could also be used to discover gene-based SNPs. The 3' ends of 94 selected genes were analyzed in 20 Indian and Chinese rhesus macaques. This effort identified 661 SNPs. Four hundred and fifty-seven of these SNPsappeared uniquely in either the Indian or Chinese rhesus macaques. A subset of those SNPs was further tested using a SNP genotyping assay against a population of 75 additional Chinese and Indian animals, as well as 4 known Chinese/Indian hybrid rhesus. Eighty-four percent of the SNPs were confirmed to show population-specificity. All of the known hybrids were detected with the Indian/Chinese SNP Assay panel.

The Indian/Chinese SNP Assay panel will be useful for animal ancestry determination, for the identification of hybrid animals and for the genetic analysis of the phenotypic differences that characterize the two populations. Further, this work validates the efficiency of using gene-based, 3' primers to discover SNPs in rhesus macaques.

## Results

### Rhesus macaque SNP discovery

We selected 20 unrelated rhesus macaques for use in SNP discovery, 10 each of alleged Indian or Chinese ancestry. All of the Chinese rhesus monkeys were imported directly from primate suppliers in China. However, neither records of capture site or breeding history were available to more specifically identify their geographic origins within Asia. Two of the Indian animals had been wild-caught in Northern India, while the remaining 8 rhesus macaques came from U.S. breeders who indicated that the animals were of Indian-descent. To validate the ancestries of all of the animals, we sequenced the *12S rRNA *portion of the mitochondrial DNAs and compared them with previously published sequences from Indian and Chinese rhesus macaques [2,4]. We also used the allele sizes of three microsatellite loci with disparate allele frequencies in Indian and Chinese populations (DX18S537, D1S548, DXS2506) to corroborate the animal ancestries [6]. Only rhesus macaques that satisfied each of these criteria for being of pure Indian or Chinese heritage were included in this study.

**Figure 1:**
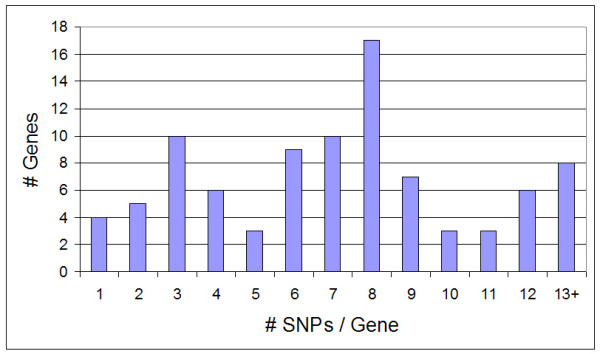
**Number of SNPs identified in the 3' end of 91 rhesus macaque genes**. SNPs were identified in 20 Chinese and Indian rhesus macaques. There were no SNPs identified in 3 genes; between one and 18 polymorphisms were found in the other 91 genes.

The 3' ends of 94 different genes were amplified and sequenced in this study, with the average amplicon length being 712 bp/gene (range: 334–905 bp). The 94 genes included at least one from every chromosome with the exceptions of 17 and Y (see Additional file 1 – Gene summary). In total we identified 661 SNPs in the rhesus macaques (see Additional file 2 – SNP identity and NCBI accession numbers). All except for three of the genes (*AR*, *CHRM4*, *IL7R*) identified at least 1 SNP, with the overall average being 7.3 SNPs/gene (Figure 1). Some genes were very polymorphic: 20 genes contained between 10 and 17 SNPs (Figure 1; see Additional file 1 – Gene summary). The overall minor allele frequency (MAF) of the SNPs was 0.127, with 281 SNPs having a MAF ≥ 0.2 in the sample population.

To assess how the SNPs identified in rhesus macaques compared with those found in the same regions of the human orthologs, we identified 10 genes that were also analyzed in a study of 48 individuals of either European and African decent [18]. The rhesus sequences contained more polymorphisms than did the human sequences, averaging 1 SNP/107 bp in the rhesus compared with 1 SNP/179 bp in humans (Table 1). None of the common SNPs in these gene regions were detected in both humans and rhesus macaques.

### Population comparison of rhesus macaque SNPs

**Figure 2:**
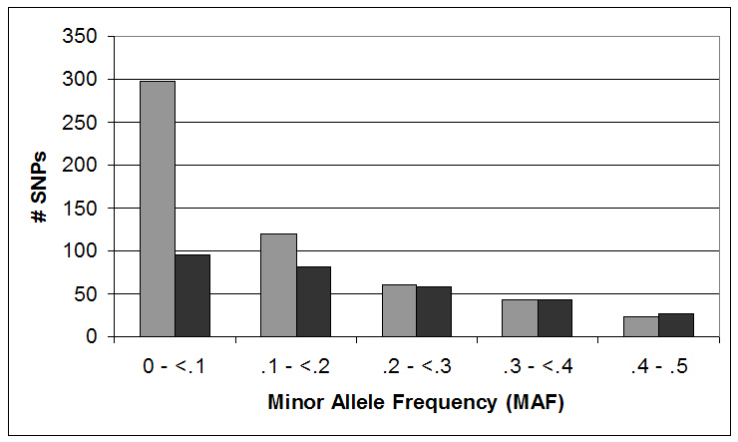
**Minor allele frequencies of SNPs in Chinese and Indian rhesus macaque populations**. The number of SNPs found in Chinese (gray) and Indian (black) rhesus macaque populations, at differing minor allele frequencies (MAF), are shown.

We analyzed the SNPs discovered within each rhesus macaque population. We identified 555 SNPs in the Chinese group, while the Indian animals had a total of 312 SNPs (Table 2; see Additional file 1 – Gene summary). The distribution of the minor allele frequencies of the SNPs within each population ranged from 0.04 to 0.5 (Figure 2). There were a higher percentage of rare alleles (MAF < 0.1) and a lower average minor allele frequency (0.15) in the Chinese animals than in the Indian rhesus (average MAF = 0.20), which is consistent with the Chinese population being more diverse.

A large number of SNPs were found exclusively in either the Indian or Chinese rhesus macaque populations. A total of 107 SNPs were unique to the Indian rhesus macaques, while 350 SNPs were only identified in the Chinese animals (Table 2; see Additional file 1 – Gene summary). Almost two thirds of the Chinese-specific SNPs were relatively rare, occurring in less than 10% of the population. However, 61 of the Chinese-specific SNPs and 39 of the Indian-specific SNPs had an allele frequency of ≥ 0.2. In some cases, the population-specific SNP allele was the major allele in that population. (For example, CD40LG:738 – the "G" allele is unique to the Indian population and occurs at 0.65 frequency.) The *MAOA *gene contained a polymorphism that was fixed in each population.

### Genotype assay of population-specific SNPs

To further explore the population-specificity of the SNPs, we genotyped 79 additional rhesus macaques at 44 of the SNPs appearing only in the Indian or Chinese groups. The animals included 28 additional Chinese-origin rhesus imported from China. The Indian rhesus included 47 Indian rhesus with well-documented breeding histories, obtained from one of 3 U.S. National Primate Research Centers (California, Yerkes and Oregon). Four unrelated animals known to be rhesus macaques hybrids based on their breeding records were also included (1/4 Chinese: 21864, 23905, 18855, 20183).

**Figure 3:**
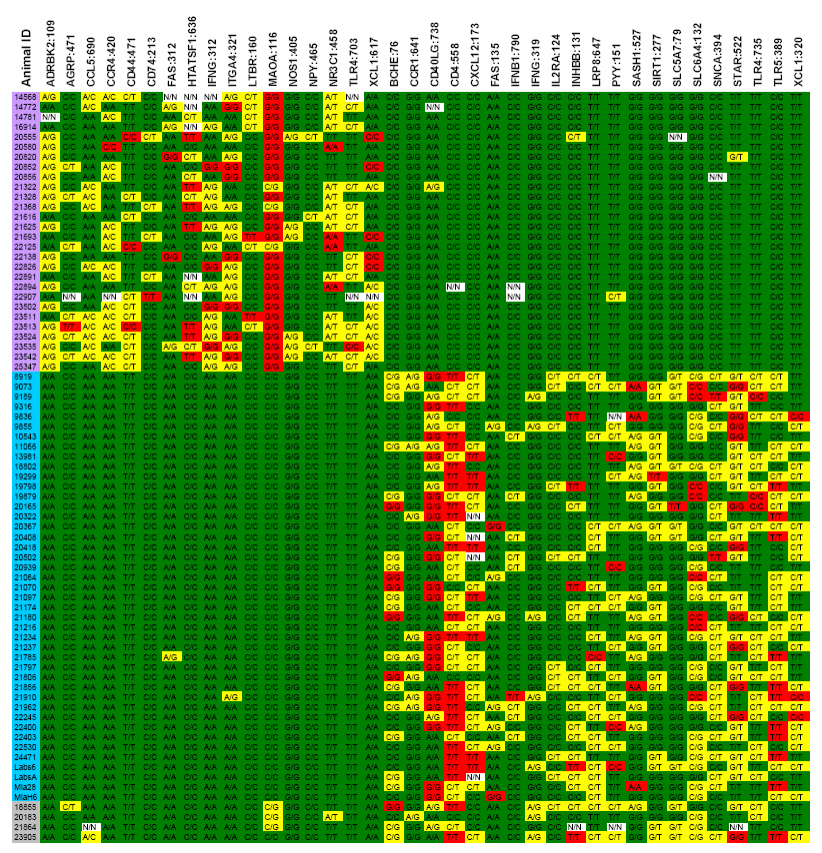
**Results of population-specific SNP Assay**. The animal identification numbers are shown on the left: Chinese rhesus are highlighted in purple, Indian rhesus in blue and documented hybrid animals are in gray. The SNP loci included are listed across the top. Genotype results are shaded as follows (based upon all animals in both populations): homozygous major alleles are in green, homozygous minor alleles are in red, heterozygous alleles are shown in yellow, and no information is indicated by white.

The 44 putative population-specific SNPs were interrogated using the Sequenom iPLEX assay [19]. Six of the SNPs (14%) were excluded when they were found to have 3 or more population exceptions, (CCR9:315, CD4:192, CD40:150, DAF:408, NOS1:216, SASH1:149). The 38 remaining SNPs are shown in Figure 3. Only three animals had exceptions in MAO:116; each of these animals was obtained from facilities in Kunming, in southwest China. One of the same rhesus macaques (21322) also contained a second "Indian-specific" allele (CD40LG:738). Three other Chinese rhesus harbored one "Indian-specific" allele (STAR:522, INHBB:131, PYY:151). Two of the Indian rhesus carried an otherwise "Chinese-specific" allele (FAS:312 and ITGA4:321).

The 4 hybrid animals included in the study carried both Chinese and Indian-specific alleles (Figure 3). All but one of the animals was heterozygous for the MAOA:116 SNP, with the exception being a 1/4 Chinese hybrid (23905), which carried 2 Indian alleles (C/C). Three other Chinese alleles appeared in this group of four animals (AGRP:471, CCL5:690, NR3C1:458).

## Discussion

This study identified a striking difference in the genome composition of Indian and Chinese rhesus macaques. There were 76% more SNPs identified in Chinese than in Indian rhesus macaques, a finding consistent with the greater mtDNA sequence and morphological diversity described for Chinese rhesus macaques [3,10,20,21]. The increased genetic heterogeneity in Chinese rhesus could be a consequence of the Chinese animals inhabiting a large geographic range and evolving more regional population substructure [3]. In addition, the reduced diversity of the Indian rhesus macaques suggests this population may have experienced an evolutionary bottleneck or expanded relatively recently from a small founder population.

A surprising finding was that 69% of the SNPs identified were unique to either Chinese or Indian rhesus macaques. If the initial cohort of animals chosen for sequencing included any misclassified or hybrid animals, then the real number of private SNPs would be even higher. However based upon both the background screening and the genotype analysis of a larger set of animals, the initial cohort appears to have correctly reported ancestries.

Thirty-eight of the 44 SNPs genotyped in an additional 75 rhesus were verified as being largely population-specific. A few animals carried one or two bases that were exceptions to the population-specificity, suggesting that some of the alleles may be present in both rhesus subtypes, but occur at very different frequencies. Six of the Chinese animals carried an otherwise "Indian" allele. Those allele exceptions (MAOA:116, STAR:522, INHBB:131, CD40LG:738, PYY:151) may be characteristic of a subpopulation of Chinese animals, such as those in western China or near the Myanmar border [3]. Alternatively, the spurious population exceptions may have resulted from the intermixing of Indian and Chinese rhesus macaques at some breeding colonies. Future studies with native populations may clarify this point.

Based upon the results of an expanded genotype assay, we predict that as many as 85% of the population-specific SNPs identified in this study will remain largely exclusive to one ancestral group when examined in additional studies. We therefore estimate that 80–85 of the SNPs identified in this study are both population-specific and occur at high frequency (MAF ≥ 0.2). These SNPs will be useful for validating rhesus macaque ancestry in an efficient manor. The overall high frequency of population-specific SNPs also suggests that a large-scale genome array of either Indian or Chinese-specific SNPs could be generated. Such an array would enable admixture studies of population-associated phenotypes.

Some of the alleles queried in the 38 SNP genotyping panel (ADBRK2:109, CD40LG:738, CD4:558) were found to deviate from Hardy-Weinberg equilibrium. The apparent under representation of particular genotypes may reflect the small number of animals sampled, population substructure, or possibly selective pressure against these, or linked, genotypes.

Based upon a comparison of 10 gene sequences that were analyzed in similar studies of rhesus macaque and human populations, it appears that the Indian and Chinese rhesus has about 1.6 times as many SNPs as the African and European human populations. Given that only 20 animals were included in this study, that estimate is likely to be conservative. The higher level of heterogeneity is not surprising, since the evolutionary history of the macaques is longer, affording more time for sequence variations to accumulate.

Some of the SNPs identified in this study may be functional, disrupting microRNA target sequences or affecting gene expression through another mechanism. Indeed, 3'UTR polymorphisms in the rhesus macaque *DAT *[22] and *TPH2 *genes [23] were shown to be associated with altered gene expression, and in the case of *TPH2*, with hypothalamic-pituitary-adrenal (HPA) axis activity. A finding from this study was that the *MAOA *gene contains alleles that significantly differ in Indian and Chinese rhesus macaques. The *MAOA *gene encodes an enzyme that contributes to the breakdown of serotonin; thus we speculate that disparate *MAOA *expression or activity may contribute to the different levels of 5-HIAA (serotonin metabolite) and the aggressive behavior that distinguish Chinese and Indian rhesus macaques [12,24].

## Conclusion

This work demonstrates that the 3' end of rhesus macaque genes is a rich source for identifying sequence polymorphisms. This study also demonstrates that there is a high frequency of SNPs that are unique to either Indian or Chinese-origin rhesus macaques. These population-specific SNPs can be used to rapidly screen and establish rhesus macaque ancestry and to identify genes that underlie phenotypes characteristic of each population.

## Methods

### Animals used in this study

Twenty unrelated rhesus macaques at the Oregon National Primate Research Center (ONPRC) were included in this study. The ten Chinese-origin rhesus used in the SNP discovery were imported from various suppliers: Laboratory Breeding Experimental Farm, Shunde, Guandong, China; Kunming Institute of Zoology, Kunming, China; Oriental Scientific Instruments of Beijing, China; Osage Research Primates, Osage Beach, MO; National Primate Center of Kunming, Kunming, China; Guandong Scientific Instruments, Guangdong Scientific Instruments & Materials Import/Export Corporation, Guangzhou, China. Two of the Indian rhesus were trapped in Northern India and exported directly to the ONPRC in 1976. The other 8 animals were obtained from: Primelabs, Inc., Hazelton Research Products, Texas Primate Center; Primate Imports, Inc.; LABS of Virginia, Yemassee VA; Scripps Clinical and Research Foundation, LaJolla, CA.

The 79 animals genotyped in the SNP assay were obtained from various sources. Twelve Chinese animals were exported from Guandong Scientific Instruments, 7 were obtained from Oriental Scientific Instruments in Beijing, 2 were from the Institute of Laboratory Animal Science in Beijing, 3 were obtained from the Kunming Institute of Zoology, and 4 were purchased from the National Primate Center of Kunming. Among the Indian rhesus macaques, 8 were obtained from the Yerkes National Primate Research Center, 7 were provided by the California National Primate Research Center, with the remaining Indian animals and hybrid animals being from the ONPRC. Three of the latter animals were captured and imported directly from Northern India in 1976.

### DNA extraction and validation of animal ancestry

Ten ml of blood was drawn from each animal in accordance with the Oregon Health and Sciences University Institutional Animal Care and Use Committee (0492-09), using universal precautions. DNA was extracted from each blood sample (QiAMP Blood Midi Kit, Qiagen, Inc.).

Two genetic analyses were used to corroborate the ancestry of each animal. First, a portion of the mtDNA *12S rRNA *locus was PCR amplified (primers F-5' ACTGGGATTAGATACCCCACT-3' and R-5'AGGGTGACGGGCGGTGTGT-3'), sequenced and compared to published Indian and Chinese rhesus macaque sequences [4]. Second, the allele sizes of three microsatellite markers (DX18S537, D1S548, DXS2506), which have differing allele frequencies in Chinese and Indian rhesus macaques, were established for each animal (Veterinary Genetics Laboratory, Davis, CA). Only rhesus macaques with mtDNA and microsatellite results supporting their documented ancestry were included in this study.

### Amplification and sequencing of DNAs

The genomics extracted from the 10 Chinese and 10 Indian rhesus macaques were used as templates for the PCR amplifications. PCR was performed as described in previously [16]. PCR products were sequenced in both directions either at Agencourt Biosciences, Inc. (Beverly, MA), or at the ONPRC using a Genetic Analyzer 3130 (Applied Biosystems, Inc.) using the amplification primers.

### SNP analysis

**Figure 4:**
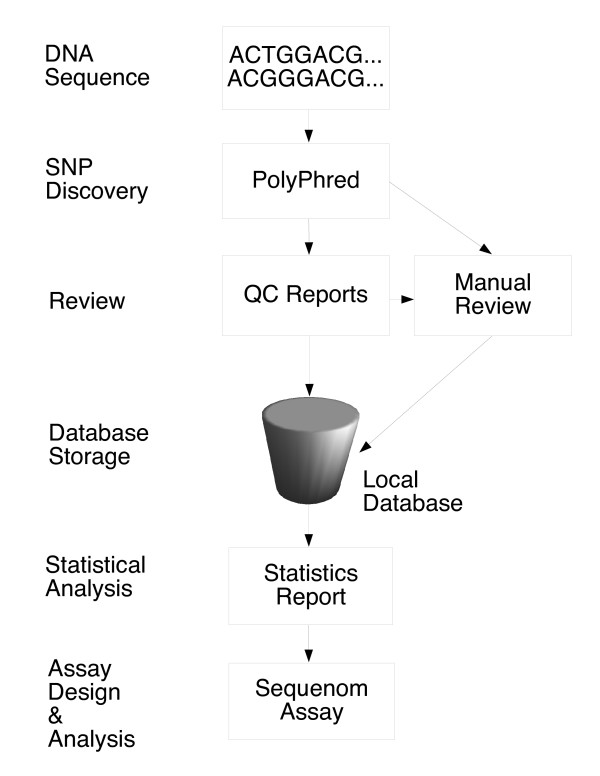
**SNP analysis workflow**. The DNA sequence output was analyzed using PolyPhred software to identify SNPs and to report the relative quality score of the SNP calls. SNPs with low quality scores or with inconsistent reports were reviewed manually. The approved SNP data was deposited into a local database for population, frequency and genotype analysis. Based upon the statistical results, some SNPs were selected for inclusion in Sequenom iPlex SNP panel.

The workflow for the DNA sequence analysis and SNP identification is outlined in Figure 4. After receipt of raw data from sequencing source, files were compiled on a Macintosh XServer at the OHSU Advanced Computing Center cluster. The Phred/Phrap/Consed package was used to perform sequence alignment [25,26]. The output sequences were aligned directly against the publish reference sequences [27], rather than being assembled into contigs first. Identification of polymorphisms on the aligned sequences was done using PolyPhred [25]. Control of these procedures was implemented in a Perl program designed for this work. Outputs from PolyPhred were then processed and loaded into a local MS-SQL-based genetics database, using a Java-based program developed for this project. In most cases, both the forward and reverse sequence reads for an individual were in agreement; in the rare case that the two sequence reads contained a difference in a single base assignment, the read with the higher PolyPhred quality score was automatically identified and entered into the database as the correct genotype for that individual. In cases where identical quality scores were attributed to a divergent base, a manual identification of the correct genotype was determined using Sequencher software (Gene Codes Corporation, Ann Arbor, MI) and visual inspection. A visual review of the sequence quality of each gene contig was also carried out before inclusion of SNPs into the final data set. A second Java program written for this project facilitated the statistical genetic calculations. It was used to calculate allele frequencies for animals in the Indian and Chinese populations, both individually and when combined; this program can also compare individuals to pre-defined populations. The verified SNPs were deposited into dbSNP [28] and the rhesus-specific database, Monkey SNP [29].

### SNP genotyping

Genotyping was performed using iPLEX reagents and protocols for multiplex PCR, single base primer extension (SBE) and generation of mass spectra, as per the manufacturer's instructions (for complete details see iPLEX Application Note, Sequenom, San Diego). Three multiplexed assays contained 28, 17, and 7 SNPs, for a total of 52 SNPs queried, of which 50 successfully generated genotyping data. Briefly, initial multiplexed PCR was performed in 5 μl reactions on 384-well plates containing 5 ng of genomic DNA. Reactions contained 0.5 U HotStar Taq polymerase (QIAGEN), 100 nM primers, 1.25× HotStar Taq buffer, 1.625 mM MgCl_2_, and 500 μM dNTPs. Following enzyme activation at 94°C for 15 min, DNA was amplified with 45 cycles of 94°C × 20 sec, 56°C × 30 sec, 72°C × 1 min, followed by a 3 minute extension at 72°C. Unincorporated dNTPs were removed using shrimp alkaline phosphatase (0.3 U, Sequenom). Single-base extension was carried out by addition of SBE primers at concentrations from 0.625 μM (low MW primers) to 1.25 μM (high MW primers) using iPLEX enzyme and buffers (Sequenom, San Diego) in 9 μl reactions. Reactions were desalted and SBE products measured using the MassARRAY Compact system, and mass spectra were analyzed using TYPER software (Sequenom, San Diego), in order to generate genotype calls and allele frequencies.

## Authors' contributions

BF and RBN conceived of and designed the study. BF selected and verified ancestry of the animals used in this study, supervised all of the data analysis, designed and analyzed the SNP genotype assay and wrote the manuscript. SLS was responsible for sequence analysis, SNP validation, annotation, integrity of the statistics and contributed to both the SNP assay design and the writing of the manuscript. HW developed algorithms for the SNP analysis and CP created the MonkeySNP website; CJD supervised their work. YJ performed some of the sequencing; ES supervised that sequencing. SLT and PA amplified and purified the PCR products. RBN identified the PCR primers, supervised the PCR amplifications and contributed to the writing of the manuscript. All authors read and approved this final manuscript.

## Supplementary Material

Additional File 1 **Gene summary**. 11 KB in size. The rhesus macaque genes included in this study are listed along with their putative chromosome location. The number of SNPs found in each population, along with the number that were unique to the population, are included. Those genes that are represented in the SNP genotype assay are also indicated. Click here for file

Additional File 2 **SNP identity and NCBI accession numbers**. 307 KB in size. This file lists the SNPs, and their associated gene, identified in this study. The NCBI STS (reference sequence) and dbSNP (SNP) accession numbers for each are shown. Click here for file

## Figures and Tables

**Table 1:** SNPs identified in the same regions of human and rhesus macaque orthologs

Gene	Human SNPs^1^	*M. mulatta *SNPs^2^
AGRP	4	6
CXCL12	4	8
DAF	3	5
IFNG	6	6
IL16	5	6
IL1A	4	9
IL2	0	1
IL8	4	8
LEP	7	2
TNF	2	12

Total	39	63

^1^From a study including 48 people of African or European heritage (SeattleSNPs).

^2^From this study.

**Table 2:** SNPs found in the 3' end of 94 rhesus macaque genes

	Chinese	Indian	Combined Populations
Total SNPs	555	312	661*
# genes	90	88	91
Frequency/gene	5.9	3.3	7
			
Unique SNPs	350	107	
# genes	83	58	
			
Unique SNPs ≥ 0.2 MAF	61	39	
# genes	29	28	

*204 SNPs were common to both populations.
