# Supplementary material for: Single nucleotide polymorphisms (SNPs) distinguish Indian-origin and Chinese-origin rhesus macaques (Macaca mulatta)
Source: BMC Genomics. 2007 Feb 7;8:43. doi: 10.1186/1471-2164-8-43 (PMC1803782; doi:10.1186/1471-2164-8-43)
Supplement: Additional File 1 — Gene summary. 11 KB in size. The rhesus macaque genes included in this study are listed along with their putative chromosome location. The number of SNPs found in each population, along with the number that were unique to the population, are included. Those genes that are represented in the SNP genotype assay are also indicated. [file 1471-2164-8-43-S1.pdf]

Additional file 1 - Gene summary

Official Symbol	Putative Chromosome	Chinese SNPs (total/unique)	Indian SNPs (total/unique)	Total SNPs	In Assay
ADRBK2	10	11/11	5/5	16	x
AGRP	20	3/3	3/3	6	x
AR	X	0/0	0/0	0	x
BCHE	2	7/5	3/1	8	x
CCL11	16	8/4	5/1	9	
CCL2	16	4/1	7/4	8	
CCL5	16	4/4	0/0	4	x
CCL8	16	8/3	5/0	8	
CCR1	2	6/5	2/1	7	x
CCR4	2	6/5	2/1	7	x
CCR6	4	8/5	4/1	9	
CCR7	16	6/3	4/1	7	
CCR8	2	2/1	2/1	3	
CCR9	2	3/3 **	0/0	3	x
CCRL1	2	6/3	5/2	8	
CD209	19	11/6	6/1	12	
CD4	11	15/12 **	5/2	17	x
CD40	10	10/4 **	6/0	10	x
CD40LG	X	8/3 *	5/1 *	9	x
CD44	14	11/10	1/0	11	x
CD47	2	3/3	1/1	4	
CD69	11	7/2	6/1	8	x
CD74	6	13/9	5/1	14	x
CFTR	3	12/9	3/0	12	
CHRFAM7A	7	7/3	5/1	8	
CHRM1	14	3/3	0/0	3	
CHRM3	1	3/2	1/0	3	
CHRM4	14	0/0	0/0	0	x
CHRM5	7	10/8	2/0	10	
CHRNA3	7	6/5	2/1	7	
CIITA	20	10/2	10/2	12	
CLN3	20	6/2	4/0	6	
CX3CR1	2	3/1	5/3	6	x
CXCL10	5	9/7	4/2	11	x
CXCL12	9	5/2	6/3	8	x
CYP11A1	7	0/0	1/1	1	
CYP17A1	9	4/3	3/2	6	
DAF	?	5/2 **	3/0	5	
FAS	9	5/5	3/3	8	x
FMR1	X	2/2	0/0	2	
FSHR	13	7/3	5/1	8	
GALC	7	6/2	7/3	9	
GBA	1	8/3 **	5/0	8	x
GLB1	2	1/0	2/1	2	
HTATSF1	X	3/3	0/0	3	x
HTR2C	X	4/3	1/0	4	
HTR3A	14	14/7	7/0	14	
IDUA	5	2/1	4/3	5	
IFNB1	15	4/1	7/4	8	x
IFNG	11	5/2	4/1	6	x

Additional file 1 - Gene summary

Official Symbol	Putative Chromosome	Chinese SNPs (total/unique)	Indian SNPs (total/unique)	Total SNPs	In Assay
IL1	12	8/3	6/1	9	
IL12B	6	3/2	2/1	4	
IL16	7	5/4	3/2	7	x
IL2	5	1/0	1/0	1	
IL2RA	9	15/11	6/2	17	x
IL6	3	1/1	0/0	1	
IL6ST	6	7/0	12/5	12	
IL7R	6	0/0	0/0	0	x
IL8	5	8/3	5/0	8	
INHBB	12	6/3	4/1	7	x
INSL6	15	1/0	2/1	2	
INSM1	10	1/0	2/1	2	
ITGA4	12	5/5	0/0	5	x
ITGAX	20	11/2	11/2	13	
ITGB2	3	7/4	3/0	7	
KAL1	X	3/2	1/0	3	
LEP	3	2/1	1/0	2	
LRP8	1	2/2	1/1	3	x
LTBR	11	11/10	2/1	12	x
MAOA	X	9/7 *	2/0 *	9	x
MAP3K5	4	3/3	0/0	3	
MPDZ	15	13/7	6/0	13	x
NDN	7	5/4	2/1	6	
NF1	16	1/1	0/0	1	
NOS1	11	7/4	4/1 **	8	x
NOS2A	16	17/17	1/1	18	
NPC1	18	3/0	3/0	3	
NPY	3	8/4	4/0	8	x
NR3C1	6	5/5	2/2	7	x
PAH	11	6/5	1/0	6	
PRDX2	19	3/0	3/0	3	
PYY	16	3/3	2/2	5	x
SASH1	4	5/4 **	6/5	10	x
SIRT1	9	5/3	3/1	6	x
SLC18A3	9	6/4	4/2	8	
SLC5A7	12	8/7	2/1	9	x
SLC6A4	16	6/3	4/1	7	x
SMARCA4	19	4/3	1/0	4	
SNCA	5	5/1	7/3	8	x
STAR	8	7/5	7/5	12	x
TLR4	15	6/4	3/1	7	x
TLR5	1	8/3	8/3	11	x
TNF	4	12/5	7/0	12	x
XCL1	1	3/2	2/1	4	x

* Fixed alleles have not been included in the unique SNP count

** Assay results using one of these SNPs indicates that it occurs in both populations
