# Supplementary material for: Single nucleotide polymorphisms (SNPs) distinguish Indian-origin and Chinese-origin rhesus macaques (Macaca mulatta)
Source: BMC Genomics. 2007 Feb 7;8:43. doi: 10.1186/1471-2164-8-43 (PMC1803782; doi:10.1186/1471-2164-8-43)
Supplement: Additional File 2 — SNP identity and NCBI accession numbers. 307 KB in size. This file lists the SNPs, and their associated gene, identified in this study. The NCBI STS (reference sequence) and dbSNP (SNP) accession numbers for each are shown. [file 1471-2164-8-43-S2.pdf]

Additional file 2 - SNP identity and accession numbers from NCBI

Official Symbol	*Gene Name*	Reference Sequence Accession #	SNP	dbSNP Accession #
ADRBK2	*adrenergic, beta, receptor kinase 2*	BV165984	ADRBK2_109	65772299
ADRBK2	*adrenergic, beta, receptor kinase 2*	BV165984	ADRBK2_136	65772300
ADRBK2	*adrenergic, beta, receptor kinase 2*	BV165984	ADRBK2_185	65772301
ADRBK2	*adrenergic, beta, receptor kinase 2*	BV165984	ADRBK2_204	65772302
ADRBK2	*adrenergic, beta, receptor kinase 2*	BV165984	ADRBK2_232	65772303
ADRBK2	*adrenergic, beta, receptor kinase 2*	BV165984	ADRBK2_305	65772304
ADRBK2	*adrenergic, beta, receptor kinase 2*	BV165984	ADRBK2_324	65772305
ADRBK2	*adrenergic, beta, receptor kinase 2*	BV165984	ADRBK2_330	66536932
ADRBK2	*adrenergic, beta, receptor kinase 2*	BV165984	ADRBK2_339	65772306
ADRBK2	*adrenergic, beta, receptor kinase 2*	BV165984	ADRBK2_346	66536933
ADRBK2	*adrenergic, beta, receptor kinase 2*	BV165984	ADRBK2_397	65772308
ADRBK2	*adrenergic, beta, receptor kinase 2*	BV165984	ADRBK2_520	65772309
ADRBK2	*adrenergic, beta, receptor kinase 2*	BV165984	ADRBK2_545	65772310
ADRBK2	*adrenergic, beta, receptor kinase 2*	BV165984	ADRBK2_624	65772312
ADRBK2	*adrenergic, beta, receptor kinase 2*	BV165984	ADRBK2_701	65772313
ADRBK2	*adrenergic, beta, receptor kinase 2*	BV165984	ADRBK2_76	65772314
AGRP	*agouti related protein*	BV686555	AGRP_107	66536934
AGRP	*agouti related protein*	BV686555	AGRP_363	66536935
AGRP	*agouti related protein*	BV686555	AGRP_380	66536936
AGRP	*agouti related protein*	BV686555	AGRP_471	66536937
AGRP	*agouti related protein*	BV686555	AGRP_531	66536938
AGRP	*agouti related protein*	BV686555	AGRP_603	66536939
BCHE	*butyrylcholinesterase*	BV211040	BCHE_198	65772317
BCHE	*butyrylcholinesterase*	BV211040	BCHE_27	66536940
BCHE	*butyrylcholinesterase*	BV211040	BCHE_393	65772319
BCHE	*butyrylcholinesterase*	BV211040	BCHE_447	65772320
BCHE	*butyrylcholinesterase*	BV211040	BCHE_464	65772321
BCHE	*butyrylcholinesterase*	BV211040	BCHE_469	65772322
BCHE	*butyrylcholinesterase*	BV211040	BCHE_538	65772324
BCHE	*butyrylcholinesterase*	BV211040	BCHE_76	65772326
CCL11	*chemokine (C-C motif) ligand 11*	BV209115	CCL11_110	65772327
CCL11	*chemokine (C-C motif) ligand 11*	BV209115	CCL11_114	65772328
CCL11	*chemokine (C-C motif) ligand 11*	BV209115	CCL11_145	65772329
CCL11	*chemokine (C-C motif) ligand 11*	BV209115	CCL11_265	66536941
CCL11	*chemokine (C-C motif) ligand 11*	BV209115	CCL11_268	66536942
CCL11	*chemokine (C-C motif) ligand 11*	BV209115	CCL11_269	66536943
CCL11	*chemokine (C-C motif) ligand 11*	BV209115	CCL11_295	65772330
CCL11	*chemokine (C-C motif) ligand 11*	BV209115	CCL11_436	66536944
CCL11	*chemokine (C-C motif) ligand 11*	BV209115	CCL11_447	66536945
CCL2	*chemokine (C-C motif) ligand 2*	BV166059	CCL2_143	65772332
CCL2	*chemokine (C-C motif) ligand 2*	BV166059	CCL2_401	65772333
CCL2	*chemokine (C-C motif) ligand 2*	BV166059	CCL2_488	65772334
CCL2	*chemokine (C-C motif) ligand 2*	BV166059	CCL2_492	65772335
CCL2	*chemokine (C-C motif) ligand 2*	BV166059	CCL2_521	65772336
CCL2	*chemokine (C-C motif) ligand 2*	BV166059	CCL2_541	65772337
CCL2	*chemokine (C-C motif) ligand 2*	BV166059	CCL2_575	65772338
CCL2	*chemokine (C-C motif) ligand 2*	BV166059	CCL2_67	65772339
CCL5	*chemokine (C-C motif) ligand 5*	BV686556	CCL5_106	66536946
CCL5	*chemokine (C-C motif) ligand 5*	BV686556	CCL5_690	66536947
CCL5	*chemokine (C-C motif) ligand 5*	BV686556	CCL5_73	66536948
CCL5	*chemokine (C-C motif) ligand 5*	BV686556	CCL5_85	66536949
CCL8	*chemokine (C-C motif) ligand 8*	BV686557	CCL8_219	66536950

CCL8	*chemokine (C-C motif) ligand 8*	BV686557	CCL8_248	66536951
CCL8	*chemokine (C-C motif) ligand 8*	BV686557	CCL8_353	66536952
CCL8	*chemokine (C-C motif) ligand 8*	BV686557	CCL8_384	66536953
CCL8	*chemokine (C-C motif) ligand 8*	BV686557	CCL8_452	66536954
CCL8	*chemokine (C-C motif) ligand 8*	BV686557	CCL8_516	66536955
CCL8	*chemokine (C-C motif) ligand 8*	BV686557	CCL8_586	66536956
CCL8	*chemokine (C-C motif) ligand 8*	BV686557	CCL8_618	66536957
CCR1	*chemokine (C-C motif) receptor 1*	BV166066	CCR1_182	65772341
CCR1	*chemokine (C-C motif) receptor 1*	BV166066	CCR1_320	65772342
CCR1	*chemokine (C-C motif) receptor 1*	BV166066	CCR1_463	65772343
CCR1	*chemokine (C-C motif) receptor 1*	BV166066	CCR1_590	65772344
CCR1	*chemokine (C-C motif) receptor 1*	BV166066	CCR1_641	65772345
CCR1	*chemokine (C-C motif) receptor 1*	BV166066	CCR1_687	65772346
CCR1	*chemokine (C-C motif) receptor 1*	BV166066	CCR1_734	66536958
CCR4	*chemokine (C-C motif) receptor 4*	BV166067	CCR4_183	65772347
CCR4	*chemokine (C-C motif) receptor 4*	BV166067	CCR4_252	65772348
CCR4	*chemokine (C-C motif) receptor 4*	BV166067	CCR4_286	65772349
CCR4	*chemokine (C-C motif) receptor 4*	BV166067	CCR4_420	65772350
CCR4	*chemokine (C-C motif) receptor 4*	BV166067	CCR4_575	65772351
CCR4	*chemokine (C-C motif) receptor 4*	BV166067	CCR4_621	65772352
CCR4	*chemokine (C-C motif) receptor 4*	BV166067	CCR4_749	65772353
CCR6	*chemokine (C-C motif) receptor 6*	BV166068	CCR6_202	65772354
CCR6	*chemokine (C-C motif) receptor 6*	BV166068	CCR6_210	65772355
CCR6	*chemokine (C-C motif) receptor 6*	BV166068	CCR6_215	65772356
CCR6	*chemokine (C-C motif) receptor 6*	BV166068	CCR6_265	65772357
CCR6	*chemokine (C-C motif) receptor 6*	BV166068	CCR6_317	65772358
CCR6	*chemokine (C-C motif) receptor 6*	BV166068	CCR6_442	65772360
CCR6	*chemokine (C-C motif) receptor 6*	BV166068	CCR6_507	65772361
CCR6	*chemokine (C-C motif) receptor 6*	BV166068	CCR6_578	65772362
CCR6	*chemokine (C-C motif) receptor 6*	BV166068	CCR6_618	65772364
CCR7	*chemokine (C-C motif) receptor 7*	BV686558	CCR7_397	66536959
CCR7	*chemokine (C-C motif) receptor 7*	BV686558	CCR7_408	66536960
CCR7	*chemokine (C-C motif) receptor 7*	BV686558	CCR7_470	66536961
CCR7	*chemokine (C-C motif) receptor 7*	BV686558	CCR7_593	66536962
CCR7	*chemokine (C-C motif) receptor 7*	BV686558	CCR7_615	66536963
CCR7	*chemokine (C-C motif) receptor 7*	BV686558	CCR7_641	66536964
CCR7	*chemokine (C-C motif) receptor 7*	BV686558	CCR7_726	66536965
CCR8	*chemokine (C-C motif) receptor 8*	BV166069	CCR8_31	66536966
CCR8	*chemokine (C-C motif) receptor 8*	BV166069	CCR8_467	65772365
CCR8	*chemokine (C-C motif) receptor 8*	BV166069	CCR8_570	65772366
CCR9	*chemokine (C-C motif) receptor 9*	BV166070	CCR9_216	65772367
CCR9	*chemokine (C-C motif) receptor 9*	BV166070	CCR9_315	65772368
CCR9	*chemokine (C-C motif) receptor 9*	BV166070	CCR9_702	65772370
CCRL1	*chemokine (C-C motif) receptor-like 1*	BV208414	CCRL1_140	65772371
CCRL1	*chemokine (C-C motif) receptor-like 1*	BV208414	CCRL1_172	65772372
CCRL1	*chemokine (C-C motif) receptor-like 1*	BV208414	CCRL1_271	65772373
CCRL1	*chemokine (C-C motif) receptor-like 1*	BV208414	CCRL1_324	65772374
CCRL1	*chemokine (C-C motif) receptor-like 1*	BV208414	CCRL1_383	65772376
CCRL1	*chemokine (C-C motif) receptor-like 1*	BV208414	CCRL1_533	65772377
CCRL1	*chemokine (C-C motif) receptor-like 1*	BV208414	CCRL1_54	65772378
CCRL1	*chemokine (C-C motif) receptor-like 1*	BV208414	CCRL1_590	65772379
CD209	*CD209 antigen*	BV166073	CD209_118	65772381
CD209	*CD209 antigen*	BV166073	CD209_183	65772382
CD209	*CD209 antigen*	BV166073	CD209_292	65772383
CD209	*CD209 antigen*	BV166073	CD209_320	65772384

Additional file 2 - SNP identity and accession numbers from NCBI

CD209	*CD209 antigen*	BV166073	CD209_501	65772386
CD209	*CD209 antigen*	BV166073	CD209_549	65772387
CD209	*CD209 antigen*	BV166073	CD209_561	65772388
CD209	*CD209 antigen*	BV166073	CD209_592	65772389
CD209	*CD209 antigen*	BV166073	CD209_604	65772390
CD209	*CD209 antigen*	BV166073	CD209_605	65772391
CD209	*CD209 antigen*	BV166073	CD209_641	65772392
CD209	*CD209 antigen*	BV166073	CD209_680	66536967
CD4	*CD4 antigen*	BV166079	CD4_122	65772393
CD4	*CD4 antigen*	BV166079	CD4_135	65772394
CD4	*CD4 antigen*	BV166079	CD4_157	65772395
CD4	*CD4 antigen*	BV166079	CD4_192	65772397
CD4	*CD4 antigen*	BV166079	CD4_194	65772398
CD4	*CD4 antigen*	BV166079	CD4_233	65772399
CD4	*CD4 antigen*	BV166079	CD4_244	65772400
CD4	*CD4 antigen*	BV166079	CD4_286	65772401
CD4	*CD4 antigen*	BV166079	CD4_390	65772402
CD4	*CD4 antigen*	BV166079	CD4_472	65772403
CD4	*CD4 antigen*	BV166079	CD4_527	65772404
CD4	*CD4 antigen*	BV166079	CD4_529	65772405
CD4	*CD4 antigen*	BV166079	CD4_53	65772406
CD4	*CD4 antigen*	BV166079	CD4_558	65772407
CD4	*CD4 antigen*	BV166079	CD4_595	65772408
CD4	*CD4 antigen*	BV166079	CD4_616	65772409
CD4	*CD4 antigen*	BV166079	CD4_86	65772410
CD40	*CD40 antigen*	BV686560	CD40_150	66536968
CD40	*CD40 antigen*	BV686560	CD40_180	66536969
CD40	*CD40 antigen*	BV686560	CD40_25	66536970
CD40	*CD40 antigen*	BV686560	CD40_292	66536971
CD40	*CD40 antigen*	BV686560	CD40_308	66536972
CD40	*CD40 antigen*	BV686560	CD40_333	66536973
CD40	*CD40 antigen*	BV686560	CD40_410	66536974
CD40	*CD40 antigen*	BV686560	CD40_417	66536975
CD40	*CD40 antigen*	BV686560	CD40_445	66536976
CD40	*CD40 antigen*	BV686560	CD40_89	66536977
CD40LG	*CD40 ligand*	BV686559	CD40LG_271	66536978
CD40LG	*CD40 ligand*	BV686559	CD40LG_272	66536979
CD40LG	*CD40 ligand*	BV686559	CD40LG_302	66536980
CD40LG	*CD40 ligand*	BV686559	CD40LG_303	66536981
CD40LG	*CD40 ligand*	BV686559	CD40LG_397	66537188
CD40LG	*CD40 ligand*	BV686559	CD40LG_398	66537189
CD40LG	*CD40 ligand*	BV686559	CD40LG_399	66537190
CD40LG	*CD40 ligand*	BV686559	CD40LG_581	66536982
CD40LG	*CD40 ligand*	BV686559	CD40LG_746	66536983
CD44	*CD44 antigen*	BV166078	CD44_240	65772411
CD44	*CD44 antigen*	BV166078	CD44_245	65772412
CD44	*CD44 antigen*	BV166078	CD44_300	65772413
CD44	*CD44 antigen*	BV166078	CD44_302	65772414
CD44	*CD44 antigen*	BV166078	CD44_332	65772415
CD44	*CD44 antigen*	BV166078	CD44_347	65772416
CD44	*CD44 antigen*	BV166078	CD44_361	65772417
CD44	*CD44 antigen*	BV166078	CD44_392	65772418
CD44	*CD44 antigen*	BV166078	CD44_471	65772419
CD44	*CD44 antigen*	BV166078	CD44_586	65772420
CD44	*CD44 antigen*	BV166078	CD44_97	65772421

CD47	*CD47 antigen*	BV208418	CD47_181	65772422
CD47	*CD47 antigen*	BV208418	CD47_516	65772423
CD47	*CD47 antigen*	BV208418	CD47_700	65772424
CD47	*CD47 antigen*	BV208418	CD47_752	65772425
CD69	*CD69 antigen*	BV166080	CD69_129	65772426
CD69	*CD69 antigen*	BV166080	CD69_229	65772427
CD69	*CD69 antigen*	BV166080	CD69_294	65772428
CD69	*CD69 antigen*	BV166080	CD69_45	65772429
CD69	*CD69 antigen*	BV166080	CD69_457	65772430
CD69	*CD69 antigen*	BV166080	CD69_504	66536984
CD69	*CD69 antigen*	BV166080	CD69_763	65772432
CD69	*CD69 antigen*	BV166080	CD69_780	66536985
CD74	*CD74 antigen*	BV686561	CD74_124	66536986
CD74	*CD74 antigen*	BV686561	CD74_138	66536987
CD74	*CD74 antigen*	BV686561	CD74_186	66536988
CD74	*CD74 antigen*	BV686561	CD74_192	66536989
CD74	*CD74 antigen*	BV686561	CD74_213	66536990
CD74	*CD74 antigen*	BV686561	CD74_247	66536991
CD74	*CD74 antigen*	BV686561	CD74_264	66536992
CD74	*CD74 antigen*	BV686561	CD74_269	66536993
CD74	*CD74 antigen*	BV686561	CD74_270	66536994
CD74	*CD74 antigen*	BV686561	CD74_311	66536995
CD74	*CD74 antigen*	BV686561	CD74_327	66536996
CD74	*CD74 antigen*	BV686561	CD74_344	66536997
CD74	*CD74 antigen*	BV686561	CD74_437	66536998
CD74	*CD74 antigen*	BV686561	CD74_452	66536999
CFTR	*cystic fibrosis transmembrane conductance regulator, ATP-binding cassette*	BV210498	CFTR_102	65772433
CFTR	*cystic fibrosis transmembrane conductance regulator, ATP-binding cassette*	BV210498	CFTR_177	65772434
CFTR	*cystic fibrosis transmembrane conductance regulator, ATP-binding cassette*	BV210498	CFTR_192	65772435
CFTR	*cystic fibrosis transmembrane conductance regulator, ATP-binding cassette*	BV210498	CFTR_215	65772436
CFTR	*cystic fibrosis transmembrane conductance regulator, ATP-binding cassette*	BV210498	CFTR_222	65772437
CFTR	*cystic fibrosis transmembrane conductance regulator, ATP-binding cassette*	BV210498	CFTR_267	65772438
CFTR	*cystic fibrosis transmembrane conductance regulator, ATP-binding cassette*	BV210498	CFTR_431	65772439
CFTR	*cystic fibrosis transmembrane conductance regulator, ATP-binding cassette*	BV210498	CFTR_581	65772440
CFTR	*cystic fibrosis transmembrane conductance regulator, ATP-binding cassette*	BV210498	CFTR_623	65772441
CFTR	*cystic fibrosis transmembrane conductance regulator, ATP-binding cassette*	BV210498	CFTR_788	66537000
CFTR	*cystic fibrosis transmembrane conductance regulator, ATP-binding cassette*	BV210498	CFTR_796	66537001
CFTR	*cystic fibrosis transmembrane conductance regulator, ATP-binding cassette*	BV210498	CFTR_92	65772444
CHRFAM7A	*CHRFAM7A*	BV686563	CHRFAM7A_106	66537002
CHRFAM7A	*CHRFAM7A*	BV686563	CHRFAM7A_117	66537003
CHRFAM7A	*CHRFAM7A*	BV686563	CHRFAM7A_172	66537004
CHRFAM7A	*CHRFAM7A*	BV686563	CHRFAM7A_185	66537005
CHRFAM7A	*CHRFAM7A*	BV686563	CHRFAM7A_242	66537006

Additional file 2 - SNP identity and accession numbers from NCBI

CHRFAM7A	*CHRFAM7A*	BV686563	CHRFAM7A_34	66537007
CHRFAM7A	*CHRFAM7A*	BV686563	CHRFAM7A_89	66537008
CHRFAM7A	*CHRFAM7A*	BV686563	CHRFAM7A_98	66537009
CHRM1	*cholinergic receptor, muscarinic 1*	BV447656	CHRM1_153	65772445
CHRM1	*cholinergic receptor, muscarinic 1*	BV447656	CHRM1_185	65772446
CHRM1	*cholinergic receptor, muscarinic 1*	BV447656	CHRM1_330	65772447
CHRM3	*cholinergic receptor, muscarinic 3*	BV210899	CHRM3_132	66537010
CHRM3	*cholinergic receptor, muscarinic 3*	BV210899	CHRM3_348	65772450
CHRM3	*cholinergic receptor, muscarinic 3*	BV210899	CHRM3_735	66537011
CHRM5	*M5 muscarinic receptor*	BV686564	CHRM5_157	66537012
CHRM5	*M5 muscarinic receptor*	BV686564	CHRM5_184	66537013
CHRM5	*M5 muscarinic receptor*	BV686564	CHRM5_224	66537014
CHRM5	*M5 muscarinic receptor*	BV686564	CHRM5_380	66537015
CHRM5	*M5 muscarinic receptor*	BV686564	CHRM5_421	66537016
CHRM5	*M5 muscarinic receptor*	BV686564	CHRM5_431	66537017
CHRM5	*M5 muscarinic receptor*	BV686564	CHRM5_659	66537018
CHRM5	*M5 muscarinic receptor*	BV686564	CHRM5_660	66537019
CHRM5	*M5 muscarinic receptor*	BV686564	CHRM5_691	66537020
CHRM5	*M5 muscarinic receptor*	BV686564	CHRM5_741	66537021
CHRNA3	*cholinergic receptor, nicotinic, alpha polypeptide 3*	BV166100	CHRNA3_100	65772451
CHRNA3	*cholinergic receptor, nicotinic, alpha polypeptide 3*	BV166100	CHRNA3_164	65772452
CHRNA3	*cholinergic receptor, nicotinic, alpha polypeptide 3*	BV166100	CHRNA3_167	65772453
CHRNA3	*cholinergic receptor, nicotinic, alpha polypeptide 3*	BV166100	CHRNA3_192	65772454
CHRNA3	*cholinergic receptor, nicotinic, alpha polypeptide 3*	BV166100	CHRNA3_23	66537022
CHRNA3	*cholinergic receptor, nicotinic, alpha polypeptide 3*	BV166100	CHRNA3_554	65772457
CHRNA3	*cholinergic receptor, nicotinic, alpha polypeptide 3*	BV166100	CHRNA3_741	66537023
CIITA	*MHC class II transactivator*	BV686565	CIITA_146	66537024
CIITA	*MHC class II transactivator*	BV686565	CIITA_178	66537025
CIITA	*MHC class II transactivator*	BV686565	CIITA_210	66537026
CIITA	*MHC class II transactivator*	BV686565	CIITA_24	66537027
CIITA	*MHC class II transactivator*	BV686565	CIITA_248	66537028
CIITA	*MHC class II transactivator*	BV686565	CIITA_286	66537029
CIITA	*MHC class II transactivator*	BV686565	CIITA_319	66537030
CIITA	*MHC class II transactivator*	BV686565	CIITA_370	66537031
CIITA	*MHC class II transactivator*	BV686565	CIITA_426	66537032
CIITA	*MHC class II transactivator*	BV686565	CIITA_61	66537033
CIITA	*MHC class II transactivator*	BV686565	CIITA_80	66537034
CIITA	*MHC class II transactivator*	BV686565	CIITA_83	66537035
CLN3	*eroid-lipofuscinosis, neuronal 3*	BV686566	CLN3_105	66537036
CLN3	*eroid-lipofuscinosis, neuronal 3*	BV686566	CLN3_160	66537037
CLN3	*eroid-lipofuscinosis, neuronal 3*	BV686566	CLN3_190	66537038
CLN3	*eroid-lipofuscinosis, neuronal 3*	BV686566	CLN3_365	66537039
CLN3	*eroid-lipofuscinosis, neuronal 3*	BV686566	CLN3_391	66537040
CLN3	*eroid-lipofuscinosis, neuronal 3*	BV686566	CLN3_400	66537041
CX3CR1	*chemokine (C-X3-C) receptor 1*	BV166144	CX3CR1_205	65772458
CX3CR1	*chemokine (C-X3-C) receptor 1*	BV166144	CX3CR1_265	65772459
CX3CR1	*chemokine (C-X3-C) receptor 1*	BV166144	CX3CR1_310	65772460
CX3CR1	*chemokine (C-X3-C) receptor 1*	BV166144	CX3CR1_321	65772461
CX3CR1	*chemokine (C-X3-C) receptor 1*	BV166144	CX3CR1_491	65772464
CX3CR1	*chemokine (C-X3-C) receptor 1*	BV166144	CX3CR1_593	65772465
CX3CR1	*chemokine (C-X3-C) receptor 1*	BV166144	CXCL10_115	65772466
CX3CR1	*chemokine (C-X3-C) receptor 1*	BV166144	CXCL10_157	65772467
CXCL10	*chemokine (C-X-C motif) ligand 10*	BV208463	CXCL10_170	65772468
CXCL10	*chemokine (C-X-C motif) ligand 10*	BV208463	CXCL10_185	65772469
CXCL10	*chemokine (C-X-C motif) ligand 10*	BV208463	CXCL10_289	65772470

Additional file 2 - SNP identity and accession numbers from NCBI

CXCL10	*chemokine (C-X-C motif) ligand 10*	BV208463	CXCL10_311	65772471
CXCL10	*chemokine (C-X-C motif) ligand 10*	BV208463	CXCL10_401	65772472
CXCL10	*chemokine (C-X-C motif) ligand 10*	BV208463	CXCL10_415	65772473
CXCL10	*chemokine (C-X-C motif) ligand 10*	BV208463	CXCL10_428	65772474
CXCL10	*chemokine (C-X-C motif) ligand 10*	BV208463	CXCL10_461	65772475
CXCL10	*chemokine (C-X-C motif) ligand 10*	BV208463	CXCL10_595	65772476
CXCL12	*chemokine (C-X-C motif) ligand 12*	BV209862	CXCL12_168	66537042
CXCL12	*chemokine (C-X-C motif) ligand 12*	BV209862	CXCL12_173	66537043
CXCL12	*chemokine (C-X-C motif) ligand 12*	BV209862	CXCL12_25	66537044
CXCL12	*chemokine (C-X-C motif) ligand 12*	BV209862	CXCL12_485	66537045
CXCL12	*chemokine (C-X-C motif) ligand 12*	BV209862	CXCL12_546	66537046
CXCL12	*chemokine (C-X-C motif) ligand 12*	BV209862	CXCL12_620	66537047
CXCL12	*chemokine (C-X-C motif) ligand 12*	BV209862	CXCL12_73	66537048
CXCL12	*chemokine (C-X-C motif) ligand 12*	BV209862	CXCL12_95	66537049
CYP11A1	*cytochrome P450, family 11, subfamily A, polypeptide 1*	BV209178	CYP11A1_150	65772477
CYP17A1	*cytochrome P450, family 17, subfamily A, polypeptide 1*	BV209179	CYP17A1_202	65772478
CYP17A1	*cytochrome P450, family 17, subfamily A, polypeptide 1*	BV209179	CYP17A1_27	66537050
CYP17A1	*cytochrome P450, family 17, subfamily A, polypeptide 1*	BV209179	CYP17A1_30	66537051
CYP17A1	*cytochrome P450, family 17, subfamily A, polypeptide 1*	BV209179	CYP17A1_408	65772479
CYP17A1	*cytochrome P450, family 17, subfamily A, polypeptide 1*	BV209179	CYP17A1_409	65772480
CYP17A1	*cytochrome P450, family 17, subfamily A, polypeptide 1*	BV209179	CYP17A1_434	66537052
DAF	*decay accelerating factor for complement*	BV166157	DAF_406	66537053
DAF	*decay accelerating factor for complement*	BV166157	DAF_54	66537054
DAF	*decay accelerating factor for complement*	BV166157	DAF_675	66537055
DAF	*decay accelerating factor for complement*	BV166157	DAF_761	66537056
DAF	*decay accelerating factor for complement*	BV166157	DAF_82	66537057
FAS	*TNF receptor superfamily, member 6*	BV686567	FAS_135	66537058
FAS	*TNF receptor superfamily, member 6*	BV686567	FAS_136	66537059
FAS	*TNF receptor superfamily, member 6*	BV686567	FAS_195	66537060
FAS	*TNF receptor superfamily, member 6*	BV686567	FAS_247	66537061
FAS	*TNF receptor superfamily, member 6*	BV686567	FAS_312	66537062
FAS	*TNF receptor superfamily, member 6*	BV686567	FAS_364	66537063
FAS	*TNF receptor superfamily, member 6*	BV686567	FAS_510	66537064
FAS	*TNF receptor superfamily, member 6*	BV686567	FAS_589	66537065
FMR1	*fragile X mental retardation 1*	BV166251	FMR1_134	65772482
FMR1	*fragile X mental retardation 1*	BV166251	FMR1_342	65772483
FSHR	*follicle stimulating hormone receptor*	BV445039	FSHR_233	65772485
FSHR	*follicle stimulating hormone receptor*	BV445039	FSHR_296	65772486
FSHR	*follicle stimulating hormone receptor*	BV445039	FSHR_320	65772487
FSHR	*follicle stimulating hormone receptor*	BV445039	FSHR_350	65772488
FSHR	*follicle stimulating hormone receptor*	BV445039	FSHR_434	65772489
FSHR	*follicle stimulating hormone receptor*	BV445039	FSHR_554	65772490
FSHR	*follicle stimulating hormone receptor*	BV445039	FSHR_566	65772491
FSHR	*follicle stimulating hormone receptor*	BV445039	FSHR_784	66537066
GALC	*galactosylceramidase*	BV686568	GALC_190	66537067
GALC	*galactosylceramidase*	BV686568	GALC_208	66537068
GALC	*galactosylceramidase*	BV686568	GALC_22	66537069
GALC	*galactosylceramidase*	BV686568	GALC_356	66537070

Additional file 2 - SNP identity and accession numbers from NCBI

GALC	*galactosylceramidase*	BV686568	GALC_370	66537071
GALC	*galactosylceramidase*	BV686568	GALC_394	66537072
GALC	*galactosylceramidase*	BV686568	GALC_402	66537073
GALC	*galactosylceramidase*	BV686568	GALC_416	66537074
GALC	*galactosylceramidase*	BV686568	GALC_436	66537075
GBA	*glucosidase, beta; acid (includes glucosylceramidase)*	BV445047	GBA_10	66537076
GBA	*glucosidase, beta; acid (includes glucosylceramidase)*	BV445047	GBA_183	65772492
GBA	*glucosidase, beta; acid (includes glucosylceramidase)*	BV445047	GBA_219	65772493
GBA	*glucosidase, beta; acid (includes glucosylceramidase)*	BV445047	GBA_336	65772494
GBA	*glucosidase, beta; acid (includes glucosylceramidase)*	BV445047	GBA_476	65772495
GBA	*glucosidase, beta; acid (includes glucosylceramidase)*	BV445047	GBA_570	66537077
GBA	*glucosidase, beta; acid (includes glucosylceramidase)*	BV445047	GBA_6	66537078
GBA	*glucosidase, beta; acid (includes glucosylceramidase)*	BV445047	GBA_68	65772496
GLB1	*galactosidase, beta 1*	BV686569	GLB1_185	66537079
GLB1	*galactosidase, beta 1*	BV686569	GLB1_383	66537080
HTATSF1	*HIV TAT specific factor 1*	BV448126	HTATSF1_346	65772503
HTATSF1	*HIV TAT specific factor 1*	BV448126	HTATSF1_636	65772504
HTATSF1	*HIV TAT specific factor 1*	BV448126	HTATSF1_767	65772505
HTR2C	*5-hydroxytryptamine (serotonin) receptor 2C*	BV166346	HTR2C_336	65772506
HTR2C	*5-hydroxytryptamine (serotonin) receptor 2C*	BV166346	HTR2C_394	65772507
HTR2C	*5-hydroxytryptamine (serotonin) receptor 2C*	BV166346	HTR2C_507	65772508
HTR2C	*5-hydroxytryptamine (serotonin) receptor 2C*	BV166346	HTR2C_643	65772509
HTR3A	*serotonin receptor 3A*	BV686570	HTR3A_140	66537081
HTR3A	*serotonin receptor 3A*	BV686570	HTR3A_218	66537082
HTR3A	*serotonin receptor 3A*	BV686570	HTR3A_328	66537083
HTR3A	*serotonin receptor 3A*	BV686570	HTR3A_343	66537084
HTR3A	*serotonin receptor 3A*	BV686570	HTR3A_348	66537085
HTR3A	*serotonin receptor 3A*	BV686570	HTR3A_362	66537086
HTR3A	*serotonin receptor 3A*	BV686570	HTR3A_384	66537087
HTR3A	*serotonin receptor 3A*	BV686570	HTR3A_397	66537088
HTR3A	*serotonin receptor 3A*	BV686570	HTR3A_420	66537089
HTR3A	*serotonin receptor 3A*	BV686570	HTR3A_428	66537090
HTR3A	*serotonin receptor 3A*	BV686570	HTR3A_446	66537091
HTR3A	*serotonin receptor 3A*	BV686570	HTR3A_548	66537092
HTR3A	*serotonin receptor 3A*	BV686570	HTR3A_67	66537093
HTR3A	*serotonin receptor 3A*	BV686570	HTR3A_678	66537094
IDUA	*alpha-L-iduronidase*	BV686571	IDUA_144	66537095
IDUA	*alpha-L-iduronidase*	BV686571	IDUA_260	66537096
IDUA	*alpha-L-iduronidase*	BV686571	IDUA_47	66537097
IDUA	*alpha-L-iduronidase*	BV686571	IDUA_487	66537098
IDUA	*alpha-L-iduronidase*	BV686571	IDUA_495	66537099
IFNB1	*interferon, beta 1, fibroblast*	BV166358	IFNB1_204	65772510
IFNB1	*interferon, beta 1, fibroblast*	BV166358	IFNB1_294	65772511
IFNB1	*interferon, beta 1, fibroblast*	BV166358	IFNB1_35	66537100
IFNB1	*interferon, beta 1, fibroblast*	BV166358	IFNB1_458	65772513
IFNB1	*interferon, beta 1, fibroblast*	BV166358	IFNB1_652	65772514
IFNB1	*interferon, beta 1, fibroblast*	BV166358	IFNB1_675	65772515

Additional file 2 - SNP identity and accession numbers from NCBI

IFNB1	*interferon, beta 1, fibroblast*	BV166358	IFNB1_789	66537101
IFNB1	*interferon, beta 1, fibroblast*	BV166358	IFNB1_790	66537102
IFNG	*interferon, gamma*	BV209304	IFNG_193	65772517
IFNG	*interferon, gamma*	BV209304	IFNG_312	65772518
IFNG	*interferon, gamma*	BV209304	IFNG_319	65772519
IFNG	*interferon, gamma*	BV209304	IFNG_391	65772520
IFNG	*interferon, gamma*	BV209304	IFNG_437	65772521
IFNG	*interferon, gamma*	BV209304	IFNG_469	65772522
IL1	*interleukin 1A*	BV686572	IL1_128	66537103
IL1	*interleukin 1A*	BV686572	IL1_309	66537104
IL1	*interleukin 1A*	BV686572	IL1_312	66537105
IL1	*interleukin 1A*	BV686572	IL1_349	66537106
IL1	*interleukin 1A*	BV686572	IL1_471	66537107
IL1	*interleukin 1A*	BV686572	IL1_479	66537108
IL1	*interleukin 1A*	BV686572	IL1_493	66537109
IL1	*interleukin 1A*	BV686572	IL1_718	66537110
IL1	*interleukin 1A*	BV686572	IL1_755	66537111
IL12B	*interleukin 12B*	BV166367	IL12B_281	65772523
IL12B	*interleukin 12B*	BV166367	IL12B_35	65772524
IL12B	*interleukin 12B*	BV166367	IL12B_4	66537112
IL12B	*interleukin 12B*	BV166367	IL12B_5	66537113
IL16	*interleukin 16*	BV166370	IL16_113	65772526
IL16	*interleukin 16*	BV166370	IL16_114	65772527
IL16	*interleukin 16*	BV166370	IL16_167	65772528
IL16	*interleukin 16*	BV166370	IL16_581	65772529
IL16	*interleukin 16*	BV166370	IL16_588	65772530
IL16	*interleukin 16*	BV166370	IL16_73	65772531
IL16	*interleukin 16*	BV166370	IL16_89	65772532
IL2	*interleukin 2*	BV686573	IL2_270	66537114
IL2RA	*interleukin 2 receptor, alpha*	BV166383	IL2RA_124	65772533
IL2RA	*interleukin 2 receptor, alpha*	BV166383	IL2RA_171	66537115
IL2RA	*interleukin 2 receptor, alpha*	BV166383	IL2RA_182	65772535
IL2RA	*interleukin 2 receptor, alpha*	BV166383	IL2RA_195	65772536
IL2RA	*interleukin 2 receptor, alpha*	BV166383	IL2RA_224	65772537
IL2RA	*interleukin 2 receptor, alpha*	BV166383	IL2RA_226	65772538
IL2RA	*interleukin 2 receptor, alpha*	BV166383	IL2RA_280	65772539
IL2RA	*interleukin 2 receptor, alpha*	BV166383	IL2RA_305	65772540
IL2RA	*interleukin 2 receptor, alpha*	BV166383	IL2RA_442	65772542
IL2RA	*interleukin 2 receptor, alpha*	BV166383	IL2RA_462	65772543
IL2RA	*interleukin 2 receptor, alpha*	BV166383	IL2RA_463	65772544
IL2RA	*interleukin 2 receptor, alpha*	BV166383	IL2RA_487	65772545
IL2RA	*interleukin 2 receptor, alpha*	BV166383	IL2RA_502	65772546
IL2RA	*interleukin 2 receptor, alpha*	BV166383	IL2RA_559	65772547
IL2RA	*interleukin 2 receptor, alpha*	BV166383	IL2RA_704	65772550
IL2RA	*interleukin 2 receptor, alpha*	BV166383	IL2RA_747	65772551
IL2RA	*interleukin 2 receptor, alpha*	BV166383	IL2RA_81	65772552
IL6	*interleukin 6*	BV445093	IL6_383	65772554
IL6ST	*interleukin 6 signal transducer*	BV208621	IL6ST_177	65772556
IL6ST	*interleukin 6 signal transducer*	BV208621	IL6ST_314	65772557
IL6ST	*interleukin 6 signal transducer*	BV208621	IL6ST_426	65772558
IL6ST	*interleukin 6 signal transducer*	BV208621	IL6ST_442	65772559
IL6ST	*interleukin 6 signal transducer*	BV208621	IL6ST_598	65772560
IL6ST	*interleukin 6 signal transducer*	BV208621	IL6ST_81	65772561
IL8	*interleukin 8*	BV166388	IL8_115	65772562
IL8	*interleukin 8*	BV166388	IL8_12	66537116

IL8	*interleukin 8*	BV166388	IL8_161	65772563
IL8	*interleukin 8*	BV166388	IL8_365	65772564
IL8	*interleukin 8*	BV166388	IL8_383	65772565
IL8	*interleukin 8*	BV166388	IL8_384	65772566
IL8	*interleukin 8*	BV166388	IL8_506	65772567
IL8	*interleukin 8*	BV166388	IL8_554	65772568
INHBB	*inhibin, beta B*	BV166390	INHBB_109	65772569
INHBB	*inhibin, beta B*	BV166390	INHBB_131	65772570
INHBB	*inhibin, beta B*	BV166390	INHBB_269	65772571
INHBB	*inhibin, beta B*	BV166390	INHBB_384	65772572
INHBB	*inhibin, beta B*	BV166390	INHBB_413	65772573
INHBB	*inhibin, beta B*	BV166390	INHBB_48	66537117
INHBB	*inhibin, beta B*	BV166390	INHBB_68	65772574
INSL6	*insulin-like 6*	BV448440	INSL6_320	65772575
INSL6	*insulin-like 6*	BV448440	INSL6_402	65772576
INSM1	*insulinoma-associated 1*	BV447777	INSM1_300	65772577
INSM1	*insulinoma-associated 1*	BV447777	INSM1_75	65772578
ITGA4	*integrin, alpha 4*	BV166398	ITGA4_118	65772579
ITGA4	*integrin, alpha 4*	BV166398	ITGA4_220	65772580
ITGA4	*integrin, alpha 4*	BV166398	ITGA4_307	65772581
ITGA4	*integrin, alpha 4*	BV166398	ITGA4_321	65772582
ITGA4	*integrin, alpha 4*	BV166398	ITGA4_68	65772583
ITGAX	*integrin, alpha X*	BV208626	ITGAX_144	65772585
ITGAX	*integrin, alpha X*	BV208626	ITGAX_216	66537118
ITGAX	*integrin, alpha X*	BV208626	ITGAX_223	65772586
ITGAX	*integrin, alpha X*	BV208626	ITGAX_281	66537119
ITGAX	*integrin, alpha X*	BV208626	ITGAX_287	66537120
ITGAX	*integrin, alpha X*	BV208626	ITGAX_291	66537121
ITGAX	*integrin, alpha X*	BV208626	ITGAX_347	65772587
ITGAX	*integrin, alpha X*	BV208626	ITGAX_410	65772588
ITGAX	*integrin, alpha X*	BV208626	ITGAX_509	65772591
ITGAX	*integrin, alpha X*	BV208626	ITGAX_537	65772592
ITGAX	*integrin, alpha X*	BV208626	ITGAX_550	65772593
ITGAX	*integrin, alpha X*	BV208626	ITGAX_616	66537122
ITGAX	*integrin, alpha X*	BV208626	ITGAX_80	65772595
ITGB2	*integrin, beta 2*	BV209318	ITGB2_286	65772596
ITGB2	*integrin, beta 2*	BV209318	ITGB2_303	65772597
ITGB2	*integrin, beta 2*	BV209318	ITGB2_319	65772598
ITGB2	*integrin, beta 2*	BV209318	ITGB2_341	65772599
ITGB2	*integrin, beta 2*	BV209318	ITGB2_357	65772600
ITGB2	*integrin, beta 2*	BV209318	ITGB2_413	65772601
ITGB2	*integrin, beta 2*	BV209318	ITGB2_62	65772602
KAL1	*Kallmann syndrome 1 sequence*	BV166406	KAL1_13	66537123
KAL1	*Kallmann syndrome 1 sequence*	BV166406	KAL1_23	66537124
KAL1	*Kallmann syndrome 1 sequence*	BV166406	KAL1_30	66537125
LEP	*leptin*	BV686574	LEP_354	66537126
LEP	*leptin*	BV686574	LEP_457	66537127
LRP8	*low density lipoprotein receptor-related protein 8, apolipoprotein e receptor*	BV209358	LRP8_185	65772613
LRP8	*low density lipoprotein receptor-related protein 8, apolipoprotein e receptor*	BV209358	LRP8_647	65772614
LRP8	*low density lipoprotein receptor-related protein 8, apolipoprotein e receptor*	BV209358	LRP8_726	66537128
LTBR	*lymphotoxin beta receptor*	BV208664	LTBR_160	65772616
LTBR	*lymphotoxin beta receptor*	BV208664	LTBR_207	65772617

Additional file 2 - SNP identity and accession numbers from NCBI

LTBR	*lymphotoxin beta receptor*	BV208664	LTBR_306	65772618
LTBR	*lymphotoxin beta receptor*	BV208664	LTBR_325	65772619
LTBR	*lymphotoxin beta receptor*	BV208664	LTBR_337	65772620
LTBR	*lymphotoxin beta receptor*	BV208664	LTBR_397	65772621
LTBR	*lymphotoxin beta receptor*	BV208664	LTBR_516	65772622
LTBR	*lymphotoxin beta receptor*	BV208664	LTBR_566	65772623
LTBR	*lymphotoxin beta receptor*	BV208664	LTBR_585	65772624
LTBR	*lymphotoxin beta receptor*	BV208664	LTBR_614	65772625
LTBR	*lymphotoxin beta receptor*	BV208664	LTBR_665	65772626
LTBR	*lymphotoxin beta receptor*	BV208664	LTBR_682	65772627
MAOA	*monoamine oxidase A*	BV166471	MAOA_100	65772628
MAOA	*monoamine oxidase A*	BV166471	MAOA_116	65772629
MAOA	*monoamine oxidase A*	BV166471	MAOA_291	65772630
MAOA	*monoamine oxidase A*	BV166471	MAOA_321	65772631
MAOA	*monoamine oxidase A*	BV166471	MAOA_336	65772632
MAOA	*monoamine oxidase A*	BV166471	MAOA_392	65772633
MAOA	*monoamine oxidase A*	BV166471	MAOA_519	65772634
MAOA	*monoamine oxidase A*	BV166471	MAOA_654	65772635
MAOA	*monoamine oxidase A*	BV166471	MAOA_711	65772636
MAP3K5	*mitogen-activated protein kinase kinase kinase 5*	BV166477	MAP3K5_159	65772640
MAP3K5	*mitogen-activated protein kinase kinase kinase 5*	BV166477	MAP3K5_411	65772641
MAP3K5	*mitogen-activated protein kinase kinase kinase 5*	BV166477	MAP3K5_487	65772642
MPDZ	*multiple PDZ domain protein*	BV447827	MPDZ_179	65772643
MPDZ	*multiple PDZ domain protein*	BV447827	MPDZ_323	65772644
MPDZ	*multiple PDZ domain protein*	BV447827	MPDZ_362	65772645
MPDZ	*multiple PDZ domain protein*	BV447827	MPDZ_368	66537129
MPDZ	*multiple PDZ domain protein*	BV447827	MPDZ_369	66537130
MPDZ	*multiple PDZ domain protein*	BV447827	MPDZ_424	65772646
MPDZ	*multiple PDZ domain protein*	BV447827	MPDZ_432	65772647
MPDZ	*multiple PDZ domain protein*	BV447827	MPDZ_487	65772648
MPDZ	*multiple PDZ domain protein*	BV447827	MPDZ_524	65772649
MPDZ	*multiple PDZ domain protein*	BV447827	MPDZ_525	65772650
MPDZ	*multiple PDZ domain protein*	BV447827	MPDZ_574	66537131
MPDZ	*multiple PDZ domain protein*	BV447827	MPDZ_71	65772651
MPDZ	*multiple PDZ domain protein*	BV447827	MPDZ_85	65772652
NDN	*necdin homolog*	BV166532	NDN_152	65772653
NDN	*necdin homolog*	BV166532	NDN_224	65772654
NDN	*necdin homolog*	BV166532	NDN_338	65772655
NDN	*necdin homolog*	BV166532	NDN_343	65772656
NDN	*necdin homolog*	BV166532	NDN_532	65772657
NDN	*necdin homolog*	BV166532	NDN_735	66537132
NF1	*neurofibromin 1 (neurofibromatosis, von Recklinghausen disease, Watson disease)*	BV211005	NF1_296	65772660
NOS1	*nitric oxide synthase 1*	BV686575	NOS1_216	66537133
NOS1	*nitric oxide synthase 1*	BV686575	NOS1_245	66537134
NOS1	*nitric oxide synthase 1*	BV686575	NOS1_295	66537135
NOS1	*nitric oxide synthase 1*	BV686575	NOS1_329	66537136
NOS1	*nitric oxide synthase 1*	BV686575	NOS1_351	66537137
NOS1	*nitric oxide synthase 1*	BV686575	NOS1_397	66537138
NOS1	*nitric oxide synthase 1*	BV686575	NOS1_405	66537139
NOS1	*nitric oxide synthase 1*	BV686575	NOS1_62	66537140
NOS2A	*nitric oxide synthase 2A*	BV209410	NOS2A_144	65772661
NOS2A	*nitric oxide synthase 2A*	BV209410	NOS2A_152	65772662
NOS2A	*nitric oxide synthase 2A*	BV209410	NOS2A_171	65772664
NOS2A	*nitric oxide synthase 2A*	BV209410	NOS2A_175	65772666

Additional file 2 - SNP identity and accession numbers from NCBI

NOS2A	*nitric oxide synthase 2A*	BV209410	NOS2A_190	66536927
NOS2A	*nitric oxide synthase 2A*	BV209410	NOS2A_207	65772667
NOS2A	*nitric oxide synthase 2A*	BV209410	NOS2A_220	65772668
NOS2A	*nitric oxide synthase 2A*	BV209410	NOS2A_250	65772669
NOS2A	*nitric oxide synthase 2A*	BV209410	NOS2A_297	65772670
NOS2A	*nitric oxide synthase 2A*	BV209410	NOS2A_298	65772671
NOS2A	*nitric oxide synthase 2A*	BV209410	NOS2A_308	65772672
NOS2A	*nitric oxide synthase 2A*	BV209410	NOS2A_339	65772674
NOS2A	*nitric oxide synthase 2A*	BV209410	NOS2A_361	65772675
NOS2A	*nitric oxide synthase 2A*	BV209410	NOS2A_465	65772676
NOS2A	*nitric oxide synthase 2A*	BV209410	NOS2A_477	65772677
NOS2A	*nitric oxide synthase 2A*	BV209410	NOS2A_90	65772678
NPC1	*Niemann-Pick C1 protein*	BV686576	NPC1_374	66537141
NPC1	*Niemann-Pick C1 protein*	BV686576	NPC1_525	66537142
NPC1	*Niemann-Pick C1 protein*	BV686576	NPC1_526	66537143
NPY	*neuropeptide Y*	BV686577	NPY_189	66537144
NPY	*neuropeptide Y*	BV686577	NPY_222	66537145
NPY	*neuropeptide Y*	BV686577	NPY_227	66537146
NPY	*neuropeptide Y*	BV686577	NPY_301	66537147
NPY	*neuropeptide Y*	BV686577	NPY_438	66537148
NPY	*neuropeptide Y*	BV686577	NPY_455	66537149
NPY	*neuropeptide Y*	BV686577	NPY_487	66537150
NPY	*neuropeptide Y*	BV686577	NPY_96	66537151
NR3C1	*nuclear receptor subfamily 3, group C, member 1*	BV166545	NR3C1_228	65772680
NR3C1	*nuclear receptor subfamily 3, group C, member 1*	BV166545	NR3C1_404	65772682
NR3C1	*nuclear receptor subfamily 3, group C, member 1*	BV166545	NR3C1_458	65772683
NR3C1	*nuclear receptor subfamily 3, group C, member 1*	BV166545	NR3C1_547	65772684
NR3C1	*nuclear receptor subfamily 3, group C, member 1*	BV166545	NR3C1_555	65772685
NR3C1	*nuclear receptor subfamily 3, group C, member 1*	BV166545	NR3C1_603	65772686
NR3C1	*nuclear receptor subfamily 3, group C, member 1*	BV166545	NR3C1_637	65772687
PAH	*phenylalanine hydroxylase*	BV210693	PAH_243	65772688
PAH	*phenylalanine hydroxylase*	BV210693	PAH_256	65772689
PAH	*phenylalanine hydroxylase*	BV210693	PAH_330	65772690
PAH	*phenylalanine hydroxylase*	BV210693	PAH_618	65772691
PAH	*phenylalanine hydroxylase*	BV210693	PAH_648	65772692
PAH	*phenylalanine hydroxylase*	BV210693	PAH_788	66537152
PRDX2	*peroxiredoxin 2*	BV209496	PRDX2_104	65772693
PRDX2	*peroxiredoxin 2*	BV209496	PRDX2_380	65772695
PRDX2	*peroxiredoxin 2*	BV209496	PRDX2_383	65772696
PYY	*peptide YY*	BV686578	PYY_113	66537153
PYY	*peptide YY*	BV686578	PYY_151	66537154
PYY	*peptide YY*	BV686578	PYY_312	66537155
PYY	*peptide YY*	BV686578	PYY_325	66537156
PYY	*peptide YY*	BV686578	PYY_351	66537157
PYY	*peptide YY*	BV686578	PYY_506	66537158
PYY	*peptide YY*	BV686578	PYY_545	66537159
PYY	*peptide YY*	BV686578	PYY_602	66537160
SASH1	*SAM and SH3 domain containing 1*	BV210813	SASH1_149	65772697
SASH1	*SAM and SH3 domain containing 1*	BV210813	SASH1_360	65772698
SASH1	*SAM and SH3 domain containing 1*	BV210813	SASH1_371	65772699
SASH1	*SAM and SH3 domain containing 1*	BV210813	SASH1_381	65772700
SASH1	*SAM and SH3 domain containing 1*	BV210813	SASH1_423	65772701
SASH1	*SAM and SH3 domain containing 1*	BV210813	SASH1_527	65772702
SASH1	*SAM and SH3 domain containing 1*	BV210813	SASH1_578	65772703
SASH1	*SAM and SH3 domain containing 1*	BV210813	SASH1_599	65772705

Additional file 2 - SNP identity and accession numbers from NCBI

SASH1	*SAM and SH3 domain containing 1*	BV210813	SASH1_612	65772706
SASH1	*SAM and SH3 domain containing 1*	BV210813	SASH1_655	65772707
SIRT1	*sirtuiun 1*	BV166718	SIRT1_266	65772709
SIRT1	*sirtuiun 1*	BV166718	SIRT1_277	65772710
SIRT1	*sirtuiun 1*	BV166718	SIRT1_489	65772711
SIRT1	*sirtuiun 1*	BV166718	SIRT1_600	65772713
SIRT1	*sirtuiun 1*	BV166718	SIRT1_62	65772714
SIRT1	*sirtuiun 1*	BV166718	SIRT1_64	66537161
SLC18A3	*solute carrier family 18 (vesicular acetylcholine), member 3*	BV686579	SLC18A3_148	66537162
SLC18A3	*solute carrier family 18 (vesicular acetylcholine), member 3*	BV686579	SLC18A3_247	66537163
SLC18A3	*solute carrier family 18 (vesicular acetylcholine), member 3*	BV686579	SLC18A3_252	66537164
SLC18A3	*solute carrier family 18 (vesicular acetylcholine), member 3*	BV686579	SLC18A3_260	66537165
SLC18A3	*solute carrier family 18 (vesicular acetylcholine), member 3*	BV686579	SLC18A3_356	66537166
SLC18A3	*solute carrier family 18 (vesicular acetylcholine), member 3*	BV686579	SLC18A3_367	66537167
SLC18A3	*solute carrier family 18 (vesicular acetylcholine), member 3*	BV686579	SLC18A3_47	66537168
SLC18A3	*solute carrier family 18 (vesicular acetylcholine), member 3*	BV686579	SLC18A3_93	66537169
SLC5A7	*solute carrier family 5 (choline transporter), member 7*	BV447953	SLC5A7_126	65772715
SLC5A7	*solute carrier family 5 (choline transporter), member 7*	BV447953	SLC5A7_163	65772716
SLC5A7	*solute carrier family 5 (choline transporter), member 7*	BV447953	SLC5A7_164	65772717
SLC5A7	*solute carrier family 5 (choline transporter), member 7*	BV447953	SLC5A7_205	65772718
SLC5A7	*solute carrier family 5 (choline transporter), member 7*	BV447953	SLC5A7_309	65772719
SLC5A7	*solute carrier family 5 (choline transporter), member 7*	BV447953	SLC5A7_415	65772720
SLC5A7	*solute carrier family 5 (choline transporter), member 7*	BV447953	SLC5A7_517	65772722
SLC5A7	*solute carrier family 5 (choline transporter), member 7*	BV447953	SLC5A7_548	65772723
SLC5A7	*solute carrier family 5 (choline transporter), member 7*	BV447953	SLC5A7_79	65772725
SLC6A4	*solute carrier family 6 , member 4*	AY083583	SLC6A4_111	65772726
SLC6A4	*solute carrier family 6 , member 4*	AY083583	SLC6A4_117	66537170
SLC6A4	*solute carrier family 6 , member 4*	AY083583	SLC6A4_132	65772727
SLC6A4	*solute carrier family 6 , member 4*	AY083583	SLC6A4_170	65772728
SLC6A4	*solute carrier family 6 , member 4*	AY083583	SLC6A4_274	65772729
SLC6A4	*solute carrier family 6 , member 4*	AY083583	SLC6A4_370	65772730
SLC6A4	*solute carrier family 6 , member 4*	AY083583	SLC6A4_468	65772731
SMARCA4	*SWI/SNF related, matrix associated, actin dependent regulator of, hromatin, subfamily a, member 4*	BV686580	SMARCA4_35	66537171
SMARCA4	*SWI/SNF related, matrix associated, actin dependent regulator of, hromatin, subfamily a, member 4*	BV686580	SMARCA4_410	66537172

SMARCA4	*SWI/SNF related, matrix associated, actin dependent regulator of, hromatin, subfamily a, member 4*	BV686580	SMARCA4_44	66537173
SMARCA4	*SWI/SNF related, matrix associated, actin dependent regulator of, hromatin, subfamily a, member 4*	BV686580	SMARCA4_61	66537174
SNCA	*alpha-synuclein*	BV686581	SNCA_251	66537175
SNCA	*alpha-synuclein*	BV686581	SNCA_318	66537176
SNCA	*alpha-synuclein*	BV686581	SNCA_38	66537177
SNCA	*alpha-synuclein*	BV686581	SNCA_394	66537178
SNCA	*alpha-synuclein*	BV686581	SNCA_429	66537179
SNCA	*alpha-synuclein*	BV686581	SNCA_477	66537180
SNCA	*alpha-synuclein*	BV686581	SNCA_52	66537181
SNCA	*alpha-synuclein*	BV686581	SNCA_586	66537182
STAR	*steroidogenic acute regulatory protein*	BV208869	STAR_199	65772733
STAR	*steroidogenic acute regulatory protein*	BV208869	STAR_247	65772734
STAR	*steroidogenic acute regulatory protein*	BV208869	STAR_290	65772735
STAR	*steroidogenic acute regulatory protein*	BV208869	STAR_388	65772736
STAR	*steroidogenic acute regulatory protein*	BV208869	STAR_460	65772737
STAR	*steroidogenic acute regulatory protein*	BV208869	STAR_461	65772738
STAR	*steroidogenic acute regulatory protein*	BV208869	STAR_463	65772739
STAR	*steroidogenic acute regulatory protein*	BV208869	STAR_486	65772740
STAR	*steroidogenic acute regulatory protein*	BV208869	STAR_522	65772741
STAR	*steroidogenic acute regulatory protein*	BV208869	STAR_555	65772742
STAR	*steroidogenic acute regulatory protein*	BV208869	STAR_616	66537183
STAR	*steroidogenic acute regulatory protein*	BV208869	STAR_99	65772743
TLR4	*toll-like receptor 4*	BV166814	TLR4_145	65772744
TLR4	*toll-like receptor 4*	BV166814	TLR4_231	65772745
TLR4	*toll-like receptor 4*	BV166814	TLR4_359	65772747
TLR4	*toll-like receptor 4*	BV166814	TLR4_430	65772748
TLR4	*toll-like receptor 4*	BV166814	TLR4_431	65772750
TLR4	*toll-like receptor 4*	BV166814	TLR4_703	65772751
TLR4	*toll-like receptor 4*	BV166814	TLR4_735	66537184
TLR5	*toll-like receptor 5*	BV166815	TLR5_112	65772752
TLR5	*toll-like receptor 5*	BV166815	TLR5_170	65772753
TLR5	*toll-like receptor 5*	BV166815	TLR5_284	65772754
TLR5	*toll-like receptor 5*	BV166815	TLR5_384	65772755
TLR5	*toll-like receptor 5*	BV166815	TLR5_389	65772756
TLR5	*toll-like receptor 5*	BV166815	TLR5_484	65772757
TLR5	*toll-like receptor 5*	BV166815	TLR5_560	65772758
TLR5	*toll-like receptor 5*	BV166815	TLR5_597	65772760
TLR5	*toll-like receptor 5*	BV166815	TLR5_604	65772761
TLR5	*toll-like receptor 5*	BV166815	TLR5_622	65772762
TLR5	*toll-like receptor 5*	BV166815	TLR5_641	65772763
TNF	*tumor necrosis factor*	BV166832	TNF_138	65772764
TNF	*tumor necrosis factor*	BV166832	TNF_189	65772766
TNF	*tumor necrosis factor*	BV166832	TNF_328	65772767
TNF	*tumor necrosis factor*	BV166832	TNF_371	65772768
TNF	*tumor necrosis factor*	BV166832	TNF_375	65772769
TNF	*tumor necrosis factor*	BV166832	TNF_480	65772770
TNF	*tumor necrosis factor*	BV166832	TNF_621	65772771
TNF	*tumor necrosis factor*	BV166832	TNF_676	65772772
TNF	*tumor necrosis factor*	BV166832	TNF_788	66537185
TNF	*tumor necrosis factor*	BV166832	TNF_796	66537186
TNF	*tumor necrosis factor*	BV166832	TNF_82	65772774

Additional file 2 - SNP identity and accession numbers from NCBI

TNF	*tumor necrosis factor*	BV166832	TNF_94	65772775
XCL1	*chemokine (C motif) ligand 1*	BV166859	XCL1_320	65772777
XCL1	*chemokine (C motif) ligand 1*	BV166859	XCL1_331	65772778
XCL1	*chemokine (C motif) ligand 1*	BV166859	XCL1_49	65772779
XCL1	*chemokine (C motif) ligand 1*	BV166859	XCL1_617	66537187
